# Towards Flexible Transparent Electrodes Based on Carbon and Metallic Materials

**DOI:** 10.3390/mi8010012

**Published:** 2017-01-04

**Authors:** Minghui Luo, Yanhua Liu, Wenbin Huang, Wen Qiao, Yun Zhou, Yan Ye, Lin-Sen Chen

**Affiliations:** 1 College of Physics, Optoelectronics and Energy & Collaborative Innovation Center of Suzhou Nano Science and Technology, Soochow University, Suzhou 215006, China; lmhsuda@yeah.net (M.L.); wbhuang@suda.edu.cn (W.H.); wqiao@suda.edu.cn (W.Q.); zyun@suda.edu.cn (Y.Z.); yanye@suda.edu.cn (Y.Y.); 2 Key Laboratory of Advanced Optical Manufacturing Technologies of Jiangsu Province & Key Laboratory of Modern Optical Technologies of Education Ministry of China, Soochow University, Suzhou 215006, China

**Keywords:** flexible transparent electrodes, flexible electronics, metal nanowire, metal grid, optoelectronics devices

## Abstract

Flexible transparent electrodes (FTEs) with high stability and scalability are in high demand for the extremely widespread applications in flexible optoelectronic devices. Traditionally, thin films of indium thin oxide (ITO) served the role of FTEs, but film brittleness and scarcity of materials limit its further application. This review provides a summary of recent advances in emerging transparent electrodes and related flexible devices (e.g., touch panels, organic light-emitting diodes, sensors, supercapacitors, and solar cells). Mainly focusing on the FTEs based on carbon nanomaterials (e.g., carbon nanotubes and graphene) and metal materials (e.g., metal grid and metal nanowires), we discuss the fabrication techniques, the performance improvement, and the representative applications of these highly transparent and flexible electrodes. Finally, the challenges and prospects of flexible transparent electrodes will be summarized.

## 1. Introduction

Flexible transparent electrodes (FTEs) are essential components for numerous flexible optoelectronic devices due to their excellent capacity for transparency and flexibility, including organic light-emitting diodes [1,2,3,4,5,6], solar cells [7,8,9,10,11,12], touch panels [13,14], and wearable devices [15]. Conventionally, transparent conductive oxides (TCO) such as the indium tin oxide films (ITO), fluorine doped tin oxide (FTO) [16], ZnO:Al (AZO) [17], and ZnO:Ga (GZO) [18,19] have governed the domain of optoelectronic devices for several decades. However, some innate drawbacks of the TCO films such as brittleness due to its ceramic nature [20] and high cost for the scarcity of materials such as indium limit the widespread use in flexible devices, where stretching, twisting, or bending are usually requested. Recently, potential alternative materials to TCO have been widely explored, including ultra-thin metallic film [21,22], carbon-based nanomaterials (e.g., carbon nanotubes (CNTs) [23,24], and graphene [25,26,27,28]), conducting polymer [29,30], and metallic materials (e.g., metal grid [7,31,32] and metal nanowires [33,34,35,36,37]). To replace conventional TCO films, new types of FTEs should have low processing costs and mechanical flexibility while maintaining a low sheet resistance (R_s_) and high optical transparency (T). As for FTEs based on conducting polymer, although the flexibility is improved, low conductivity and T limit their optoelectronic performance, and stability in ambient atmosphere is not good enough. Commonly, CNTs with percolating networks cannot process a low R_s_ and a high T simultaneously. The low conductivity of the CNTs remains the main limiting factor of the overall conductivity. For example, to achieve an R_s_ of the CNTs based FTE less than 10 Ω□^−1^, the T will decrease drastically because the required thickness of CNTs exceeds 100 nm [38]. There remain challenges to improve the electrical conductivity of CNT-based FTEs.

Monolayer graphene only absorbs 2.3% of visible light and can sustain 4% strain with negligible cracking [39,40]. The theoretical R_s_ of the graphene is as low as 30 Ω□^−1^ [41]. However, the R_s_ of synthesized graphene usually exceeds several hundred Ω□^−1^ using different synthesis methods (e.g., epitaxial grown graphene on silicon carbide, chemical vapor (CVD) deposited on Cu catalysts), due to lower quality graphene with polycrystalline structures and plenty of defects [42,43].

Metallic based FTEs constructed from random networks of nanowires or regular metal grids regarded as another potential alternative to TCO, due to their better R_s_-T performance than other alternatives. The metal nanothough networks can obtain a FTE with R_s_ of 2 Ω□^−1^ at a transmittance of 90% [44]. In addition, the fabrication of metal structures coincides with printing and roll-to-roll technology, which reduces the cost for mass-production of FTEs significantly. However, the stability of the metal-based FTEs needs to be investigated further.

In this review, we provide a summary of recent advances in emerging FTEs and related flexible optoelectronic devices, mainly focusing on works reported in the past three years. For early work on FTEs, readers can refer to the review by Hecht et al. [45] and Ellmer et al. [46]. Carbon-based nanomaterials and metallic nanomaterials are promising to replace the dominance of the TCO films due to their superior performance, which will be the focus of our review. Then, we discuss the fabrication techniques, the performance improvement, and the representative applications of these FTEs. The challenges and prospects of the FTEs will eventually be summarized.

## 2. Currently Emerging Materials

Among those emerging alternatives, carbon-based materials and metallic materials are considered promising candidates for next-generation FTEs, due to their high mechanical flexibility paralleling good optical transparency and electrical conductivity. The material properties, combined with low material costs and fabrication techniques, make these emerging materials very attractive for FTEs.

### 2.1. One-Deminsional CNT-Based Nanomaterials

Carbon nanotubes (CNTs) have been evaluated and verified as one of the future FTEs relying on their remarkable characteristics. In the past several years, transparent, conductive, and flexible CNT-based FTEs have been investigated widely, involving many applications (e.g., OLED and supercapacitor). They can be fabricated using different methods [47,48,49], including wet and dry processing, which could be further exploited by combination with the roll-to-roll process. Both single-walled CNTs (SWCNTs) and multiwalled CNT (MWCNT)-based FTEs have been fabricated via solution approaches [24,50,51]. In detail, the SWCNT powders made from the CVD process are centrifuged to remove large conglomerations, before blending into an SWCNT solution. After that, the FTEs can be fabricated by various methods with the solution, including spray coating, dip-coating, and infiltration. Wu et al. have reported a simple process for the fabrication of FTEs using pure SWCNTs [52]. Low sheet resistance of 30 Ω□^−1^ was obtained with 90% transmittance. However, owing to the SWCNTs tendency of twining during the CVD process, only a small part of them can be efficiently used, which increases the production costs.

**Figure 1:**
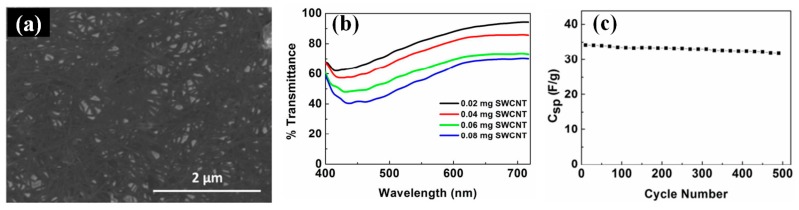
(**a**) SEM image of a SWCNT thin film with a sheet resistance of 75 Ω□^−1^; (**b**) Optical transmittance characteristics of the full devices with respect to total SWCNT weight in both electrodes; (**c**) Cycle performance of flexible supercapacitors at a current density of 1.25 A/g. Reproduced with permission from Reference [49]. Copyright 2014, American Chemical Society.

To explore cost-effective method, Feng et al. employed a straightforward roll-to-roll process to fabricate flexible and stretchable MWCNT films as FTEs, where the CNTs possess excellent performance with low sheet resistance (208 Ω□^−1^) and high transmittance (90%) [47]. In the work, CNT arrays are obtained by batch growth. Lin et al. reported polyaniline composite films incorporated with aligned MWCNTs, which were fabricated using an easy electrodeposition process [48]. Additionally, Recep et al. reported FTEs based on SWCNTs (Figure 1a), which exhibited an optical transmittance of 82% with 0.02 mg SWCNTs (Figure 1b). The supercapacitors fabricated by the SWCNT FTEs showed good capacity retention (94%) upon cycling over 500 times (Figure 1c) [49].

### 2.2. Two-Dimensional Graphene-Based Nanomaterials

Graphene, a two-dimensional carbon allotrope, becomes a popular material for transparent electrodes due to the excellent performance [25,53,54]. Generally, the R_s_ of synthesized graphene usually exceed several hundred Ω□^−1^ [42,43]. To improve the photoelectric performance, combing graphene with other conductors is an effective method.

**Figure 2:**
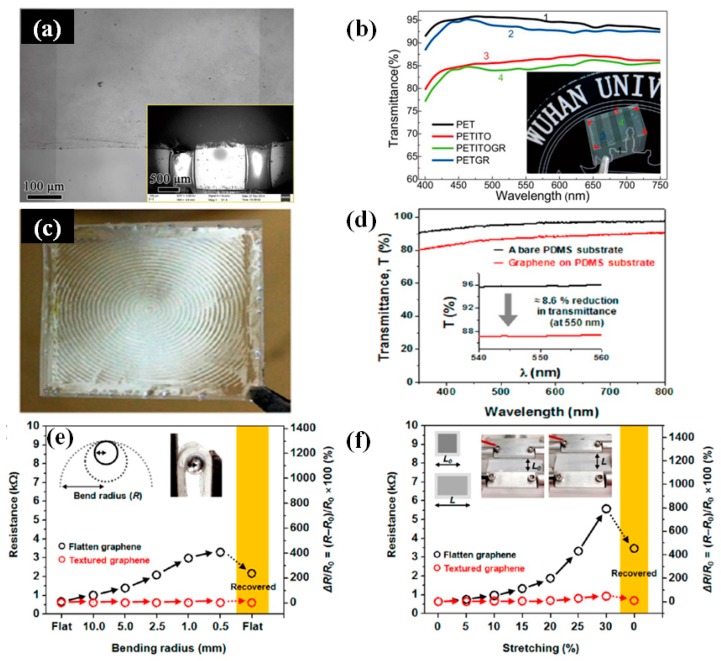
(**a**) Optical image of graphene/ITO hybrid electrode; (**b**) Transmittance spectra of the samples on PET substrate. (**a**,**b**) Reproduced with permission from Ref. [55]. Copyright 2016, Springer Nature; (**c**) Photograph of a graphene/PDMS free-standing film; (**d**) Transmission spectra of a flat PDMS substrate (black) and multilayered graphene on a flat PDMS substrate (red); (**e**) bending radius and (**f**) tensile strains (insets: actual test images for a graphene/PDMS film); (**c**–**f**) Reproduced with permission from Reference [57]. Copyright 2016, American Chemical Society.

**Figure 3:**
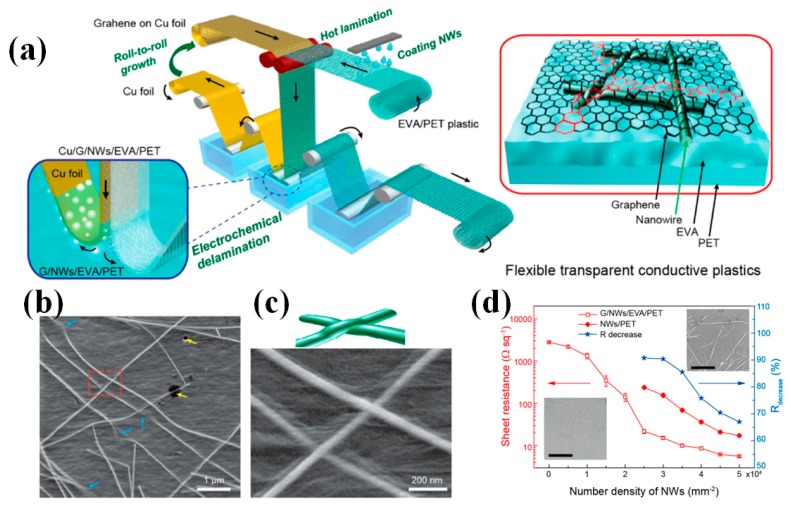
(**a**) Schematic diagram of the fabrication process includes coating of metal nanowires on polymer substrate (EVA/PET), hot-press lamination with graphene/Cu foil, delamination of graphene and Cu foil by electrochemical bubbling method, and the reuse of Cu foil to grow graphene by a continuous chemical vapor deposition system. The detailed structural schematic of the hybrid film labeled in the red cycle shows that nanowires are partly embedded into the EVA substrate and fully encapsulated by monolayer graphene film; (**b**) SEM image of the monolayer graphene; (**c**) Enlarged side-view SEM image of the hybrid film of graphene and AgNWs; (**d**) Sheet resistance versus number density of AgNWs for pure AgNW film and graphene/AgNW hybrid film. Reproduced with permission from Reference [26]. Copyright 2015, American Chemical Society.

Liu et al. prepared the graphene/ITO flexible hybrid transparent electrode, which showed excellent mechanical and optoelectronic characteristics [55]. Figure 2a shows the optical image of the graphene/ITO hybrid electrode. The SEM image shows the surface of ITO bridges on left and right sides as well as the graphene film upon them. Figure 2b is the transmittance spectra of the samples on the PET substrate. Besides, combination of graphene with metal materials can significantly improve the optoelectronic performance. The metal materials and the graphene offer extra conductive paths for each other. Qiu et al. reported the combination of the metal grid with graphene oxide films via a facile, green, and room-temperature method [56]. The excellent photoelectric properties with the sheet resistance of 18 Ω□^−1^ and the transmittance of 80% can be obtained. Hong et al. demonstrated omnidirectionally stretchable and transparent graphene electrodes with good mechanical durability and performance reliability (Figure 2c) [57]. The multilayered graphene based FTE exhibits a transmittance of 87.1% at 550 nm (Figure 2d). Figure 2e,f show that sheet resistances of the graphene/PDMS film systematically monitored under various bending and stretching conditions, respectively. The textured graphene/PDMS film sustains its electrical properties when the film is folded with a bending radius (r) as small as r = 0.5 mm or stretched up to 30% of the tensile strain. Deng et al. developed a full roll-to-roll production of FTEs based on metal NWs (AgNWs and CuNWs) encapsulated by a monolayer graphene film, as shown in Figure 3a–c [26]. The R_s_ of the encapsulated NWs film show a 60%–90% decrease relative to the pure one (Figure 3d), which is due to the graphene conduction channel. Meanwhile, the encapsulated film processes lower R_s_ than pristine graphene because metal NWs provide additional conduction channels.

In addition to the graphene-based hybrid FTEs, doped graphene-based FTEs also exhibit good performance [41,58]. Bae et al. demonstrated roll-to-roll production and wet-chemical doping of predominantly monolayer 30-inch graphene films grown by CVD [41]. The doped graphene based FTEs have sheet resistances as low as ~125 Ω□^−1^ with 97.4% optical transmittance. Park et al. found that AuCl_3_ doping on graphene could improve the conductivity and shift the work function of the graphene FTEs, which results in improved power conversion efficiency of the OPV devices [58].

### 2.3. Metallic Materials

As mentioned above, the progress in carbon-based FTEs is exciting, but the R_s_ is still not low enough. For the realization of the next generation of flexible and large-area electronics, further improvement in R_s_-T performance is required. Metal-based FTEs have gained prominence in both academic and industrial use in flexible electronics. Metal-based FTEs can be constructed from random distribution metallic NWs works [33,34,35,36,37] to regular metal grids [7,31,32], which have demonstrated great potential in optical transparency, electrical conductivity, and mechanical flexibility.

#### 2.3.1. Random Distribution Metallic NWs as FTEs

Most metals have excellent electrical conductivity due to their high free-electron density. To utilize metal materials in FTEs, high transparency should be satisfied. Among common metallic nanomaterials, silver nanowires (AgNWs) are commerciogenic due to the excellent electrical conductivity and high transmittance in a broad wavelength range [33,34,35,36,37].

The fabrication of AgNW FTEs has been explored by many researchers in various fabrication processes [59], which can achieve a low R_s_ (8–50 Ω□^−1^) with a high transmittance (80%–98%). However, metal NW-based FTEs suffer from degradation of the R_s_-T performance because of high junction resistances, which have been systematical investigated by several groups [60,61]. Mutiso et al. demonstrated that a higher NW aspect ratio could decrease the R_s_ while keeping the T a constant [61]. To obtain R_s_ ≤ 10 Ω□^−1^ at T = 90%, the aspect ratio should exceed 800 at a given junction resistance of 2 KΩ. Commonly, the strategies to reduce junction resistance involve bulk heating [62], plasmonic treatment [63], and chemical modifications [64], which tend to damage the plastic substrate and are not suitable for flexible applications. Therefore, other additional processes are introduced to produce hybrid structure electrodes. For example, Xiong et al. exhibited electroless-welding of an AgNW network coated with conductive ion gel that significantly reduces junction resistance between AgNWs, while its effect on the T of the FTEs (R_s_ of 8.4 Ω□^−1^ at T = 86%) is negligible [65].

In addition, the material of AgNWs exhibits the limitation of high surface roughness, which leads to low carrier transport. Currently, AgNW-based composite materials have become promising materials as FTEs, exhibiting excellent optical and electric properties [34,66]. Lee et al. reported a percolating network of AgNWs with densities above the percolation threshold integrated into graphene as hybrid FTEs [67]. The hybrid structure can reduce R_s_ down to 33 Ω□^−1^ with a transmittance of 94%. Other research groups demonstrated the combination of an AgNW network with graphene or graphene oxide could enhance electrical conductivity and decrease surface roughness [68,69,70]. Xu et al. mixed Ag NWs with graphene oxide and obtained Ag-doped graphene fibers, whose electrical conductivity increased from 4.1 × 10^4^ S/m to 9.3 × 10^4^ S/m, exhibiting a 330% enhancement factor [71]. Meanwhile, chemical and thermal stabilities also can be further improved. It is obvious that the composite materials enhance electrical properties of FTEs by decreasing junction resistance or providing new conductive pathways.

**Figure 4:**
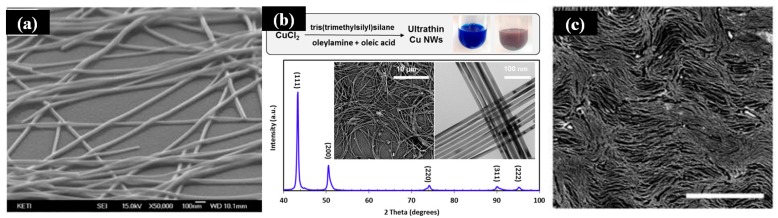
(**a**) SEM image of AgNWs. Reproduced with permission from Reference [34]. Copyright 2016, American Chemical Society; (**b**) Synthesis of ultrathin copper nanowires. Reproduced with permission from Reference [72]. Copyright 2015, American Chemical Society; (**c**) SEM image of monolayer AuNWs on PDMS substrate. Reproduced with permission from Reference [73]. Copyright 2016, American Chemical Society.

Not merely AgNWs, copper NWs (CuNWs) and gold NWs (AuNWs) are promising candidates for FTEs, due to the similarly excellent conductivity (Figure 4a–c) [34,72,73]. Cui et al. have reported a new synthetic approach for obtaining ultrathin high-quality CuNWs (Figure 4b), where oleylamine is used as a coordinating ligand [72]. Maurer et al. have demonstrated that ultrathin AuNWs can be synthesized at room temperature [73]. Triisopropylsilance (TIPS, 2 mL) is added into the solution after the gold salt was completely dissolved. The AuNWs will format standing for two days until the color turns from yellow into dark-red (Figure 4c). A high transparency of 79% can be achieved and maintained over 80 stretching cycles.

#### 2.3.2. Metal Grids

The randomly dispersed network in the whole scale cannot be used immediately as prepared. Subsequently, patterning processes are necessarily adopted depending on different device architectures. In particular, metal grid FTEs offer advantages over other NW FTEs due to the nature of artificial design. For example, the electrical and optical properties can be managed by modulating the grid pitch, width, and thickness.

**Figure 5:**
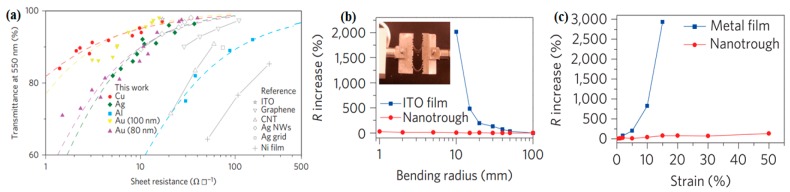
(**a**) Sheet resistance versus optical transmission (at 550 nm) for copper, gold, silver, and aluminum nanotrough networks, described by percolation theory. The performances of device-grade ITO, CNTs, graphene, silver nanowires (NWs), silver grid, and nickel thin films are shown for comparison; (**b**) R_s_ versus bending radius for bendable transparent electrodes consisting of gold nanotrough networks or ITO films on 178-μm-thick PET substrates; (**c**) R_s_ versus uniaxial strain for a stretchable transparent electrode consisting of gold nanotrough networks on 0.5-mm-thick PDMS substrate. Reproduced with permission from Ref. [44]. Copyright 2013, Nature Publishing Group.

Uniform metal grids as FTEs fabricated by many processes, including nano-patterning techniques such as photolithography, crackle, electroless plating, nano-transfer printing, and electrospinning [74,75,76,77,78]. Almost all of these processes involve deposition of a metal film onto a template to form inter-connected network excluding junction resistance. In contrast to the limited material choices for NWs, a variety of metallic materials can be exploited in different applications. Cui et al. employed electrospun and metal deposited technique to generate nanotrough networks. Correspondingly, the width and thickness of the networks are 420 nm and 80–100 nm, respectively. The new kind FTEs exhibited both superior optoelectronic performance (R_s_ of 2 Ω□^−1^ at 90% transmission) (Figure 5a) and remarkable mechanical flexibility (Figure 5b,c) [44]. The R_s_-T performance achieved is much better than that of TCO, carbon-based materials, or solution processed mental NWs networks.

In another work, Han et al. proposed the cracked TiO_2_ gel film as a template to make Ag networks [75]. The Ag networks with diameters of 1–2 μm, and widths of 4–100 μm, exhibited good electro-optical properties. The transmittance ranges from 82% to 45%, and correspondingly the R_s_ ranges from 4.2 Ω□^−1^ to 0.5 Ω□^−1^. To further improve the R_s_-T performance of the NWs based FTEs, Hsu et al. introduced a mesoscale metal-wire concept in conjunction with NWs [78]. The mesoscale metal-wire networks show the extraordinary R_s_-T performance with the R_s_ being 0.36 and the T being 92%. However, fabricating metal grid FTEs usually employs the physical deposition of metal materials involving thermal evaporation or sputtering, which requires expensive vacuum-based processing. Therefore, the fabrication process is not simple and cost-effective.

Recently, electrohydrodynamic (EHD) jet printing employed to fabricate metal grid FTEs [31,79]. Lee et al. fabricated FTEs by thermal pressing of metal lines, which was provided by EHD jet printing [31]. The excellent properties have been demonstrated with the sheet resistance of 0.5 Ω□^−1^ at the transmittance of 80%. Seong et al. have reported a method of fabricated FTEs by EHD jet printing. Sheet resistance of 1.49 Ω□^−1^ can be achieved by printing the Ag mesh on the convex glass [79]. Another route utilized to fabricate FTE by using inject printing [80]. Mohl et al. obtained grid meshes by inject printing and subsequent chemical copper plating [32]. The printed metal grids from reactive ink plated with copper can create high performance FTEs. The achieved R_s_ is 10 Ω□^−1^ and transmittance is 80%.

**Figure 6:**
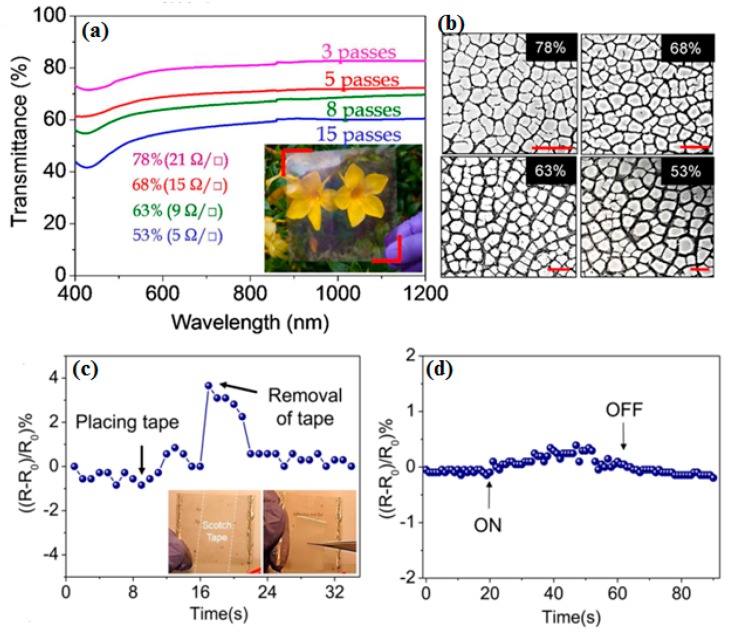

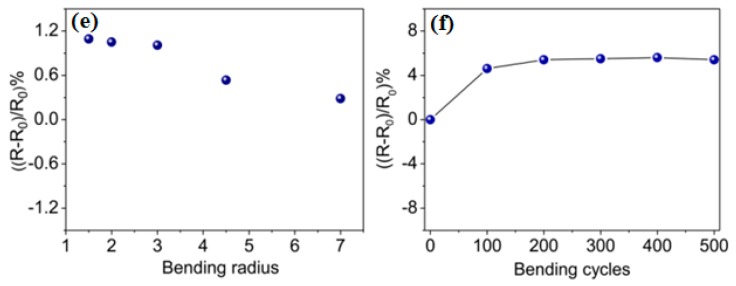
(**a**) Specular optical transmittance of Ag mesh prepared from crackles of different widths. The sheet resistances of the samples are mentioned in parentheses. Photograph of the prepared TCE with 78% transmittance at 550 nm is shown as an inset. (**b**) Optical micrographs of Ag mesh of different widths. Scale bar is 1 mm. Relative variations in the resistance of the Ag mesh during (**c**) the scotch tape adhesion test, (**d**) sonication test, (**e**) bending to different radii, and (**f**) 500 bending cycles with a radius of 1.5 mm. The photographs in the inset in (**a**) show the scotch tape pasted over the Ag mesh and while peeling off. Reproduced with permission from Reference [82]. Copyright 2015, American Chemical Society.

A novel fabrication method via hybrid printing technique has been demonstrated for FTEs with embedded metal grids [81]. Cui et al. developed high-resolution metal mesh as FTEs by nanoimprinting technology [14]. The silver nanoparticle inks embedded into the metal mesh, where the metal mesh was fabricated by the roll-to-roll progress. Low-cost fabrication is the most important advantage of this new approach. Moreover, high performance could be obtained with the low sheet resistance of 0.69 Ω□^−1^ at 88% transparency. Most important is that the fabrication cost can be further decreased by exploiting their compatibility with printing technologies, attributing to the efficient use of material, a simple fabrication process, and easy scalability to large scale. In addition, Kiruthika et al. employed the roll and spray coating methods to fabricate the FTEs by a simple solution process using crackle lithography [82]. A transmittance of 78% and sheet resistance of ~20 Ω□^−1^ can be obtained (Figure 6a). Figure 6b shows the optical micrographs of Ag meshes with different widths on PET substrate. Figure 6c–f exhibits the mechanical stability of the metallized Ag in the crackle network. The mesh was subjected to 500 bending cycles with 1.5 mm radius, the change in the resistance remained within 5%.

**Figure 7:**
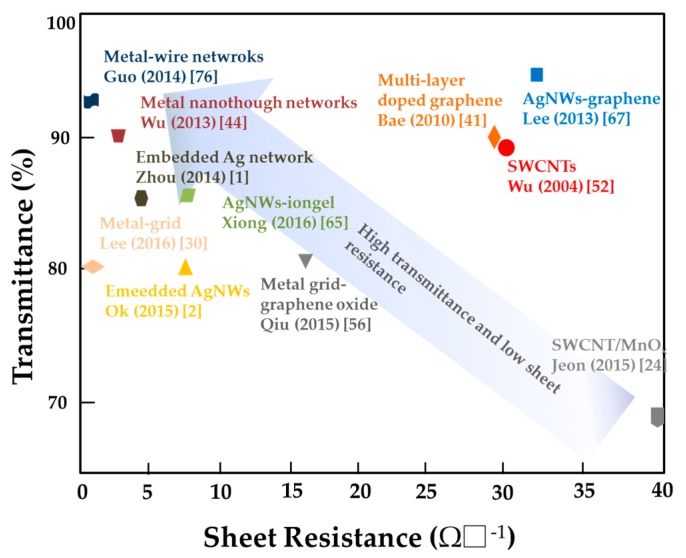
Sheet resistance/Transmission values of FTEs reported in recent works.

Finally, some typical values of sheet resistance and transmission of the novel FTEs up to date are summarized in Figure 7. Great progress has made in FTEs towards lower sheet resistance and high transmittance.

## 3. Applications

Based on the excellent mechanical compliance, high transmittance, and electric conductivity, the FTEs can be utilized in many essential applications, including supercapacitor, OLED, solar cell, and touch panel [45,46]. Different performances are needed for different applications, due to the requirement of technical indexes [60]. To satisfy the requirement of touch screens, the R_s_ of FTE should range from 100 Ω□^−1^ to 1000 Ω□^−1^, a T exceeds 85% combined with low haze. The FTEs must have the R_s_ ~ 10 Ω□^−1^ for T > 90% to provide solar cells and OLEDs. In addition, for use of FTEs in flexible optoelectronics, several requirements must be met, including the reduced haze value for the optical transparency, and ultra-smooth surface of the electrode to avoid the disconnection problem. Finally, a simple, low-cost, and large-scale process in fabricating FTEs is a necessity for commercial applications.

**Figure 8:**
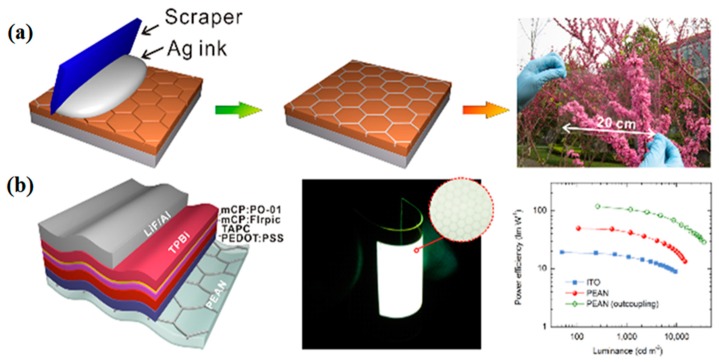
(**a**) Schematic illustration of the fabrication process of an embedded Ag network on PET (PEAN) and its optical image; (**b**) Device structure and performance of flexible white OLED using PEAN anode. Reproduced with permission from Reference [1]. Copyright 2014, American Chemical Society.

To function as an FTE in OLEDs, the metal-based electrodes must alleviate the leakage current by planarizing its surface. Zhou et al. investigated embedded Ag-grid FTEs as the anode of OLED, which exhibits a power efficiency of 106 lm·W^−1^ at 1000 cd·m^−2^ (Figure 8) [1].

**Figure 9:**
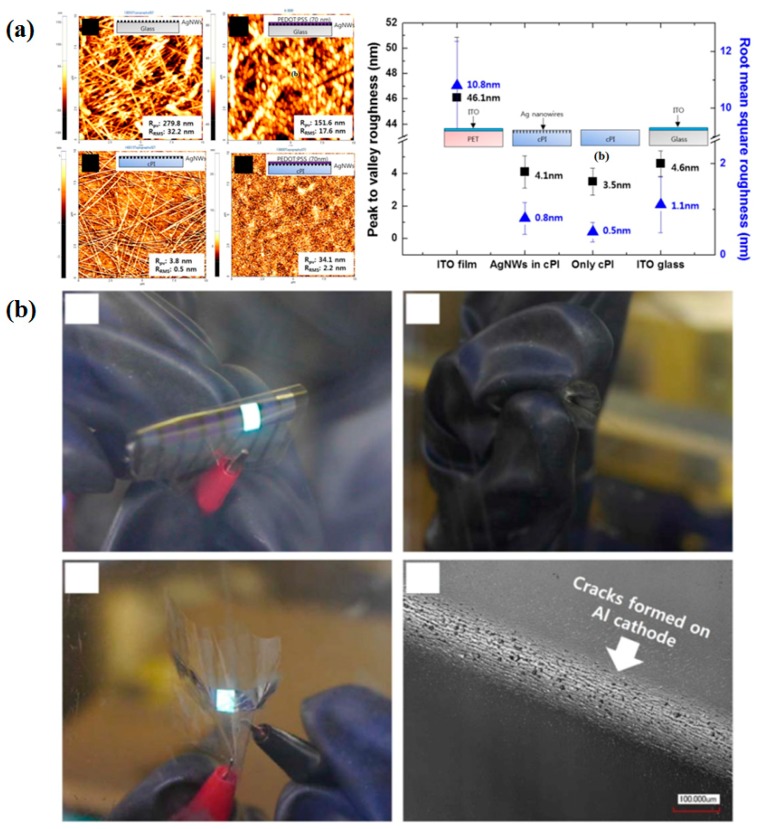
(**a**) Comparison of morphologies by AFM analyses and a diagram comparing the roughness of various samples; (**b**) Bending stability of the flexible OLEDs based on AgNWs-cPI composite electrodes. Reproduced with permission from Reference [2]. Copyright 2015, Macmillan Publishers Limited.

Liu et al. reported a composite electrode developed from poly (3,4-ethylenedioxythiophene): poly (styrenesulfonate) (PEDOT: PSS) and AgNWs with a template stripping method [3]. The efficiency of the resultant OLEDs improved by 25% compared to that of traditional PEDOT: PSS FTEs, attributing to the reduction of surface roughness and the improvement of electric conductivity. Ok and co-worker developed an ultra-thin and smooth FTE by embedding AgNWs in a colorless polymide (cPI) [2]. The AgNWs-cPI composite electrode exhibited a T > 80%, a low R_s_ of 8 Ω□^−1^, and ultra-smooth surfaces comparable to glass (4.1 nm), as shown in Figure 9a. The OLEDs fabricated from such composite electrodes showed a stable performance with a luminance reduction of <3% after 10 repeated bendings at a radius of 30 μm (Figure 9b).

For applications in display devices and touch screens, high visibility is needed. Haze property is a vital value to the display industry. Ag networks that are fabricated by cracked template and AgNWs solution processed methods [75,83] can survive multiple finger touchings. The low R_s_ (<10 Ω□^−1^) and the large area fabrication are rapidly improving in the large format touch panels market. Currently, iV-touch can offer large projected capacitive touch panels from 21.5″ to 55″ with stunning touch performance [84].

Similar to the case of OLEDs, the application of NW networks and metal grids as the TCEs employed in functional organic solar cells. In order to achieve the required R_s_ (<50 Ω□^−1^) and T (>90%), constructing hybrid structured FTEs is a promising technology. Singh et al. demonstrated solution processed solar cells employing AgNW film binding with a 40 nm thin overlayer of sputtered ZnO as FTEs [85]. The ZnO overlayer is used to increase adhesion between AgNW and ZnO/buffer layer and to interconnect the AgNWs junctions. More recently, Li et al. integrated embedded Ag grids and conducting polymer hybrid electrodes into a perovskite-based photovoltaic cell and demonstrated an ultrathin flexible device delivering a power conversion efficiency of 14.0% [7]. Wu et al. demonstrated AgNW grids with multi-length scaled structures as FTEs for an organic solar cell [86]. A power conversion efficiency of 9% was achieved for the organic solar cell devices.

**Figure 10:**
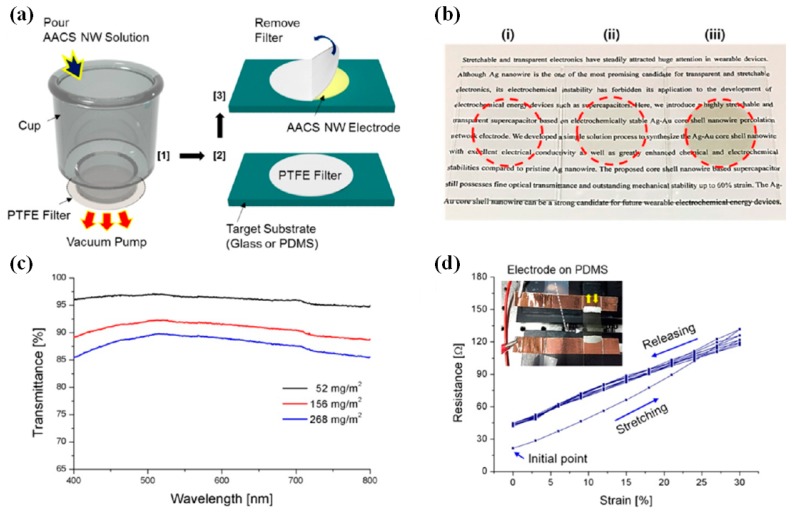
Fabrication and characterization of Ag–Au core–shell NW-based electrodes. (**a**) Schematic illustration of the electrode fabrication through vacuum filtration and transfer method; (**b**) Digital image of as-prepared transparent Ag–Au core–shell NW network electrodes at various sheet resistances; (**c**) Optical transmittance of the transparent electrodes at various areal NW densities; (**d**) Strain-dependent electrical resistance of Ag–Au core–shell NW electrode on an elastic PDMS substrate. Reproduced with permission from Reference [89]. Copyright 2016, American Chemical Society.

Transparent supercapacitors have been proposed in the past decade, which function both as a current collector to transport the electrons, and an active material to store electrochemical energy. For the application in supercapacitors, Lin et al. has synthesized polyaniline composite film incorporated with aligned MWCNTs through an easy electrodeposition process [48]. The conductive films are sufficiently used to fabricate transparent, flexible, and efficient supercapacitors with a maximum specific capacitance of 233 F/g at a current density of 1 A/g. Cai et al. demonstrated that the electrochemical stability of Ag grid FTEs can be enhanced by coating one layer of PEDOT:PSS [87]. The film sustained an optical modulation and a specific capacitance of 87.7% and 67.2% at 10 A·g^−1^, respectively. Cheng et al. reported hybrid electrodes composed of PEDOT:PSS and Ag-grids prepared via inject printing [88]. The hybrid structures not only compensated for the shortcomings of the single materials but also fully combined their advantages. The comparatively high Ct (capacitance of the supercapacitor) and Csc (capacitance of the electrode) of the supercapacitor based on PEDOT: PSS (three layers)/Ag grid electrodes were 1.13 mF·cm^−2^ and 4.52 mF·cm^−2^, respectively. In addition, Lee et al. have introduced a highly flexible and transparent supercapacitor based on electrochemically stable Ag-Au core-shell (AACS) nanowire percolation network electrode (Figure 10) [89]. Figure 10c confirms that the AACS NW networks possess superior optical transparency, exceeding 85% in the entire range of the visible wavelength. Figure 10d shows strain-dependent electrical resistance of Ag–Au core–shell NW electrode on an elastic PDMS substrate.

The various applications make carbon-based and metal-based FTE one of the most promising materials in optoelectronic devices. Considering the progress and the ongoing efforts on FTEs, there is no doubt that performance of these FTE-based flexible devices (OLED, OSC, supercapacitor, touch screen) can be further improved, and the new applications can served to an amazing degree.

## 4. Conclusions and Challenges

In conclusion, a comprehensive overview on recent developments and achievements in carbon-based and metal-based FTEs and related flexible optoelectronic devices is provided in this review. Carbon-based materials and metallic materials are promising to dominate the FTEs, especially hybrid FTEs exhibiting superior properties. Although outstanding performances—including low sheet resistance, high transmittance, and good mechanical properties—have been demonstrated, there are still many restrictions hindering large-scale fabrication. In addition, more exploration in the microcosmic aspect and the fundamental theories are needed. The present FTEs already play a significant role in many optical and electric applications, which urges more studies to improve the performance (stretchability, flexibility, transmittance, stability, and electric conductivity) from the material synthesis to device fabrication.
